# Myosin Post‐Translational Modifications Associated With Critical Illness Myopathy

**DOI:** 10.1111/apha.70240

**Published:** 2026-06-18

**Authors:** Fernando Ribeiro, Bruno Di Geronimo, Nicola Cacciani, Anna Widgren, Yvette Hedström, Anselmo S. Moriscot, Peter M. Kasson, Shina C. L. Kamerlin, Jonas Bergquist, Lars Larsson

**Affiliations:** ^1^ Center for Molecular Medicine (CMM) Karolinska Institutet Stockholm Sweden; ^2^ Department of Anatomy, Institute of Biomedical Sciences University of São Paulo São Paulo Brazil; ^3^ School of Chemistry and Biochemistry Georgia Institute of Technology Atlanta Georgia USA; ^4^ Department of Clinical Sciences, Comparative Medicine Swedish University of Agricultural Sciences Uppsala Sweden; ^5^ Analytical Chemistry and Neurochemistry, Department of Chemistry for Life Sciences Uppsala University Uppsala Sweden; ^6^ Department of Cell and Molecular Biology Uppsala University Uppsala Sweden; ^7^ Department of Chemistry Lund University Lund Sweden

**Keywords:** critical care, liquid chromatography–tandem mass spectrometry, mechanical ventilation, muscle contraction, skeletal muscle

## Abstract

**Background:**

Critical illness myopathy is a common and devastating consequence of critical care, causing dramatic loss of muscle mass and function in intensive care unit patients. Functional deficits often exceed the loss in muscle mass and myosin content. However, the mechanisms underlying the loss of force and emergence of myosin‐expressing non‐force‐generating fibers remain elusive.

**Methods:**

Myosin dysfunction was investigated in six intensive care unit patients exposed to a 12‐day mechanical ventilation and immobilization period using mass spectrometry‐based proteomics and molecular dynamics simulations.

**Results:**

Previous single muscle fiber analyses revealed decreased fiber size and specific force from the 1st to the 12th days in all patients. A subset of myosin‐expressing fibers exhibiting a complete loss of contractile function was identified in three of the patients despite similar atrophy levels (~30%, *p* < 0.05) after 12 days. All fibers had decreased specific force after 12 days of mechanical ventilation, but 9% to 21% of the fibers were non‐force generating. The decline in specific force was linked to 27 post‐translational myosin modifications, including oxidation, ubiquitination, acetylation, and methylation. Molecular dynamics simulations indicated oxidation‐induced rigidity of the myosin head, predicted to compromise the flexibility of the actin‐binding and converter domains. Non‐force‐generating fibers exhibited a unique proteomic signature predicted to enhance myosin motor domain exposure and rigidity.

**Conclusion:**

In addition to muscle wasting and myosin loss, abnormal myosin post‐translational modifications contribute to muscle weakness in ICU patients with CIM, including the development of muscle fibers incapable of generating contractile force.

## 1 Introduction

Mechanical ventilation is a lifesaving intervention frequently used in modern critical care, but mechanical ventilation is also associated with complications related to lung injury, acquired myopathies, and cognitive dysfunction. The dramatic muscle wasting and compromised force‐generating capacity of remaining contractile material negatively impact the rehabilitation of intensive care unit (ICU) patients and weaning off mechanical ventilation, resulting in prolonged ICU care with negative consequences for patient quality of life, mortality/morbidity, and health care costs (for refs. see [1]).

The loss of muscle mass and the preferential loss of the molecular motor protein myosin contribute to the decline in force generation capacity (maximum force normalized to muscle fiber cross‐sectional area, or specific force) in limb muscles [1, 2, 3]. In the diaphragm, the loss in specific force is not caused by a preferential loss of myosin, while myosin post‐translational modifications negatively affect force‐generation capacity [4, 5]. The preferential myosin loss in limb muscles is a relatively late phenomenon in mechanically ventilated and immobilized ICU patients, that is, the hallmark of the Critical Illness Myopathy (CIM), which is preceded by myosin post‐translational modifications negatively affecting the specific force in limb muscles in a similar way as in the diaphragm [6].

In experimental studies, we have previously observed an increasing proportion of non‐force‐generating diaphragm muscle fibers in rats mechanically ventilated from 6 h to 14 days. In control diaphragm muscle fibers, all single muscle fibers generated normal specific force, while 7%, 22%, and 37% were non‐force‐generating in animals immobilized and mechanically ventilated for 6 h to 4 days, 5–8 days, and 9–14 days, respectively [4]. In a more recent prospective clinical study, mechanically ventilated neuro‐ICU patients were followed for 12 days, during which six serial muscle biopsies were performed for transcriptome profiling. Additionally, contractile properties at the single muscle fiber level were measured in the first and final biopsy. In five of the 10 patients who survived 12 days of mechanical ventilation, between 9% and 21% of the measured fibers did not generate any force upon maximum calcium activation despite significant myosin expression [2].

Thus, non‐force‐generating fibers may substantially impair limb and respiratory muscle function in long‐term immobilized and mechanically ventilated ICU patients; however, the underlying mechanisms remain elusive. We hypothesize that the non‐force‐generating fibers have unique myosin modifications distinguishing them from control and force‐generating fibers from long‐term mechanically ventilated ICU patients. This hypothesis was supported by mass spectrometry‐based proteomics and molecular dynamics simulation data.

## 2 Materials and Methods

For further details, see Appendix.

### 2.1 ICU Patients

Six neuro‐ICU patients (IDs #626, #629, #632, #639, #646, and #662) from a previous study [2] were included in this investigation. All patients exhibited central nervous system injuries and their anthropometric characteristics and medical histories have been previously reported [2].

### 2.2 Muscle Biopsies

Biopsies of the tibialis anterior (TA) muscle of patients were obtained on the 1st (D1) and 12th (D12) day of ICU hospitalization using the percutaneous conchotome method [2].

### 2.3 Single Muscle Fiber Size and Specific Force

Muscle bundles were dissected from the TA muscle and permeabilized for measurements of single fiber size and contractile properties. Skinned single muscle fibers were carefully isolated and attached to force transducer connectors in the setup apparatus (custom‐built by our group). Representative fiber's absolute force (calculated as the difference between maximal isometric force and resting tension), cross‐sectional area (CSA), and specific force (absolute force normalized to CSA) were determined as the average values obtained from measurements of approximately 10 to 20 fibers per patient.

### 2.4 Mapping Myosin PTMs With LC‐MS/MS‐Based Proteomics

Twenty‐six muscle fibers isolated from five out of six ICU patients' biopsies (patient ID#662 excluded due to the lack of muscle biopsy) were used for liquid chromatography–mass spectrometry (LC–MS/MS) proteomics. The samples were distributed as follows: ICU_D1 (*n* = 4), ICU_D12 force‐generating (*n* = 11), and ICU_D12 non‐force‐generating (*n* = 11) were used for liquid chromatography–mass spectrometry (LC–MS/MS) proteomics. Myosin protein content was separated on a 12% SDS‐PAGE, and Coomassie‐stained bands corresponding to myosin heavy chain (~223 kDa) were excised, in‐gel protein digested, and the peptides analyzed by LC–MS/MS for identification of PTMs.

The acquired RAW data files were analyzed with the Proteome Discoverer 1.4.0.288 (Thermo Fisher Scientific) software using the SEQUEST HT (University of Washington) search engine against proteins from Homo Sapiens in the UniProtKB/SwissProt database downloaded June, 2022.

### 2.5 Molecular Dynamics (MD) Simulations

Human Myosin‐7 (UniProt ID:P12883; PDB ID:4DB1) [7, 8] constituted by two MYH7 chains (A and B) was selected as the model for our computational analysis. This choice builds on prior studies that have used this structure in modeling studies in order to investigate post‐translational modifications, residue interaction networks, electrostatic effects, and disease‐associated mutations in β‐cardiac (type I) myosin (see e.g., refs. [9, 10, 11]).

The high‐resolution X‐ray structure described above was modeled to reproduce the derived systems “ICU_D1”, “ICU_D12”, and “ICU_D12_NF” by incorporating PTMs, loop reconstruction and addition of ATP molecules, while keeping the crystallographic water molecules (Figures S1 and S2). The specific PTMs introduced in each system were as follows: ICU_D1 included oxidized positions N589, D752, and H753; ICU_D12 featured oxidations at K86, D89, H97, Y162, Y164, H491, F494, N589, D752, and H753, as well as methylation at K757; and ICU_D12_NF included oxidation at H97. MD simulations were carried out using AMBER24 and AmberTools [7] using the ff19SB [12] and GAFF2 force fields [13]. The three systems were initially energy‐minimized *in vacuo* [14] and subsequently solvated in a truncated octahedral box using TIP3P [15] water molecules, with counterions added to ensure charge neutrality. Three replicas of 500 ns conventional MD simulations were then conducted for each system. Trajectory analysis was carried out using the CPPTRAJ module and Matplotlib [16].

### 2.6 Statistical Analysis

Data analysis was performed by using Excel, Prism, and R. The Shapiro–Wilk test was used to assess data normality. One‐way analysis of variance (ANOVA) followed by Tukey's post hoc tests or Kruskal–Wallis's followed by Dunn's post hoc tests was used for multiple comparisons between groups. Values are presented as means ± standard deviations unless stated otherwise. Statistical significance was accepted as *p* < 0.05.

## 3 Results

**FIGURE 1:**
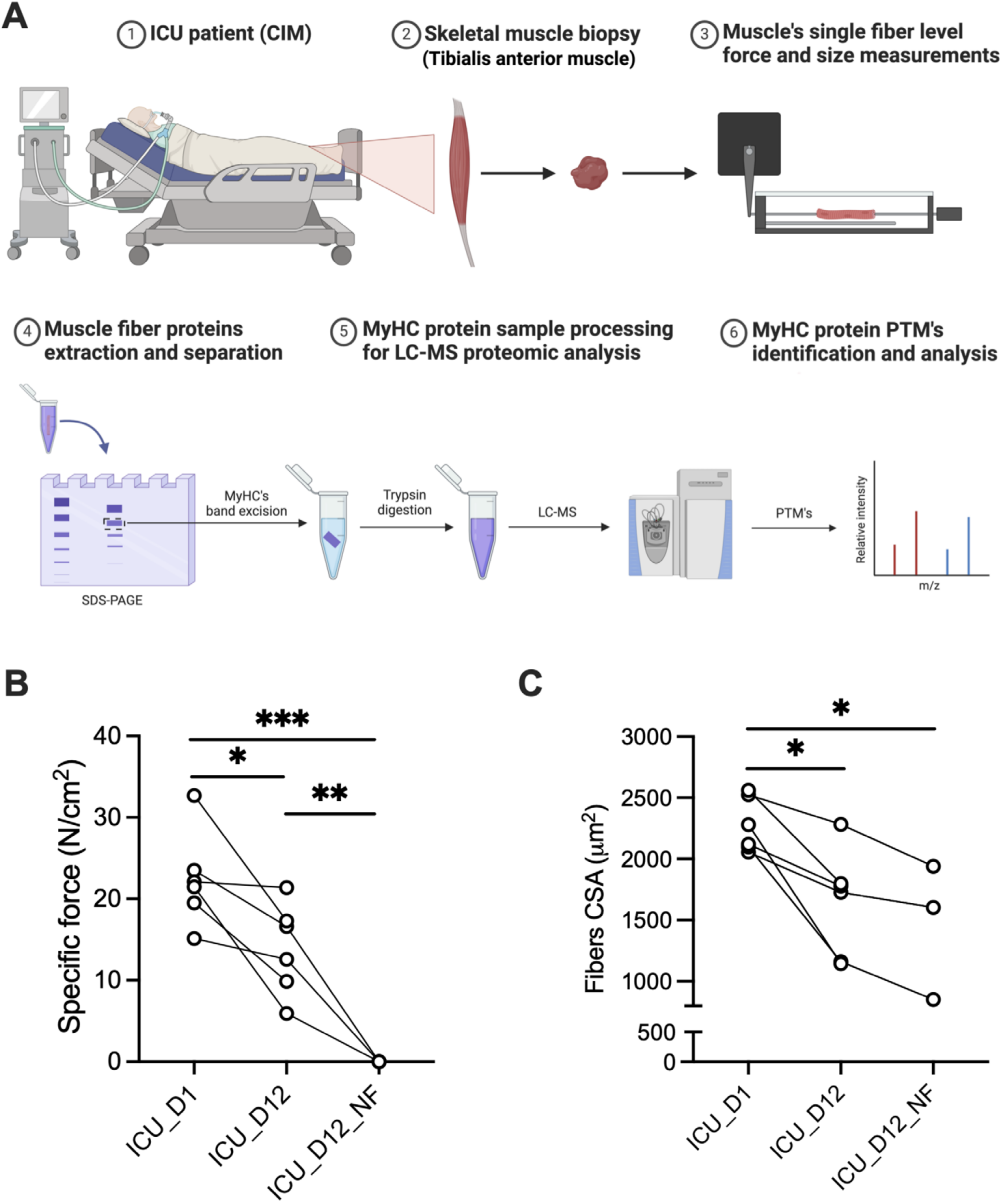
Investigating the role of PTMs on myosin as a regulatory molecular mechanism of the skeletal muscle weakness severity during critical illness myopathy. (A) Schematic illustration of the experimental design employed in this study to investigate the impact of PTMs on myosin protein structure and function in muscle weakness during critical illness myopathy. (B) ICU patient's TA muscle specific force (i.e., the absolute force normalized to the fiber's CSA), and (C) fiber size (where each point represents the mean values, defined as the average specific force and fiber CSA measurements of 10–20 fibers, per subject, *n* = 3–5 patients). The data presented for ICU_D1 and ICU_D12 groups' muscle fiber‐specific force and size originate from previously published work [2], used for comparison with ICU_D12_NF group. Data are presented as mean ± SD. *p < 0.05; **p < 0.001; and ***p < 0.0001 denote statistically significant differences among groups according to One‐way ANOVA followed by Tukey's post hoc test analysis. Graphical illustration was created with Biorender (biorender.com).

It is hypothesized that differential regulatory control of myosin protein structure by distinct PTMs represents a molecular mechanism underlying the modulation of skeletal muscle weakness severity in ICU patients. To test this hypothesis, TA muscle biopsies were obtained from six neuro‐ICU patients followed longitudinally for 12 days during immobilization and controlled mechanical ventilation previously described in detail [2]. Single muscle fiber size, maximum force, and specific force (maximum force normalized to fiber CSA) previously characterized on the 1st and 12th day of immobilization and mechanical ventilation were herein used for proteomic analysis. LC–MS/MS‐based proteomics was employed to examine myosin PTMs, followed by MD simulations for modeling the impact of PTMs on myosin protein structure and function in response to long‐term ICU exposure (Figure 1A).

### 3.1 ICU‐Induced Muscle Weakness and Wasting

**FIGURE 2:**
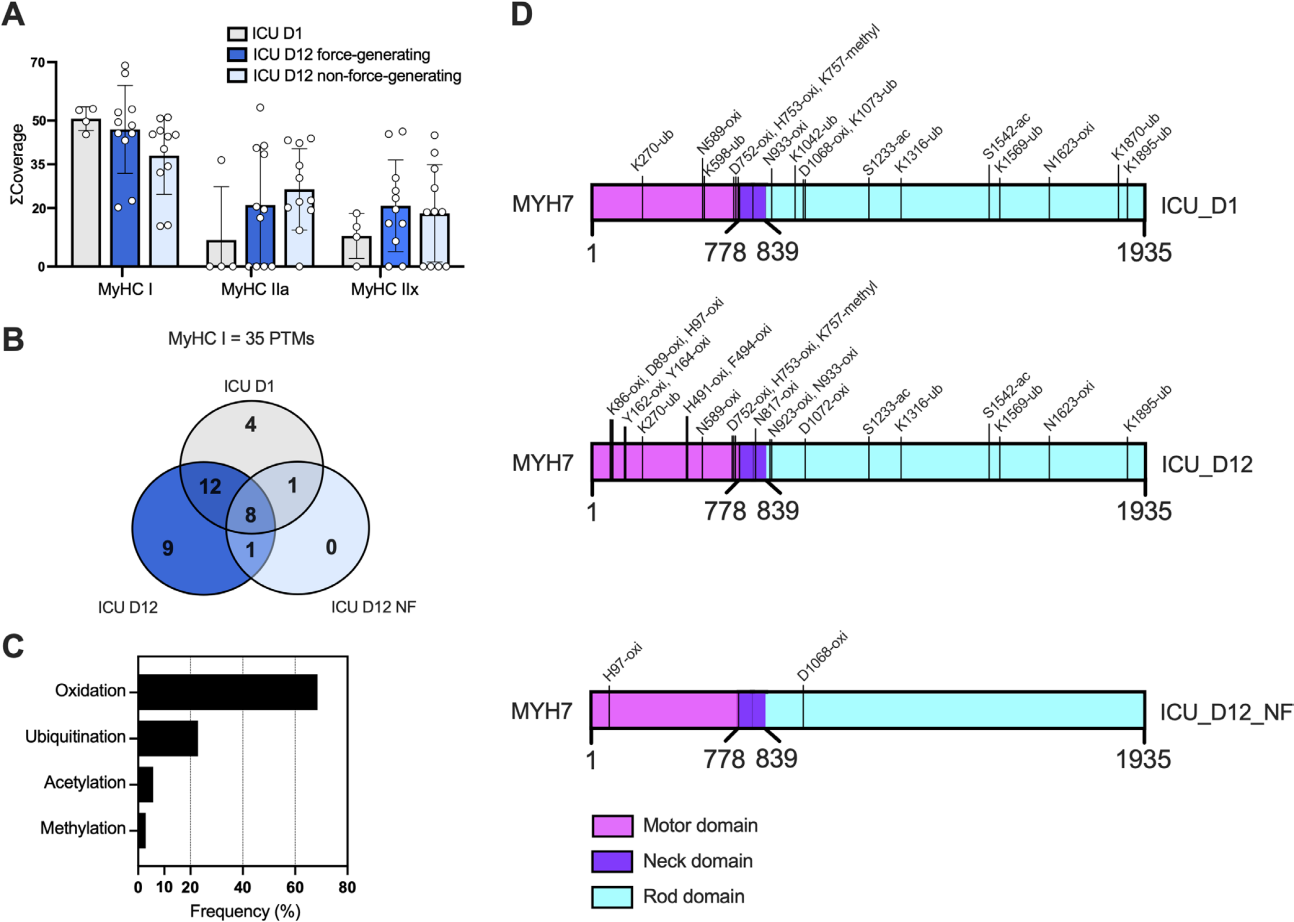
Post‐translational modifications found in the tibialis anterior slow‐twitch fibers of critically ill patients after 12 days of ICU hospitalization. (A) Total coverage of myosin heavy chain protein isoforms isolated from the tibialis anterior muscle biopsies subjected to LC–MS/MS‐based proteomic analysis. (B) Venn diagram representing the amount of post‐translational modifications identified and their distribution in each group. (C) Relative frequency of distribution of post‐translational modification per class found in the predominant MyHC type I isoform. (D) MYH7 2D model representation depicting the localization of post‐translational modifications present in the patient's muscle fibers following 1–12 days of ICU hospitalization. Data are presented as mean ± SD, *n* = 4–11.

To investigate the negative impact of ICU treatment on skeletal muscle structure and function, we examined the TA muscle fibers, whose contractile properties and size have previously been characterized at the single muscle fiber level [2]. As previously reported, a significant decline in muscle fiber size and specific force was observed at day 12 compared with day 1 (see Figure 1B,C) [2]. Strikingly, in three out of six ICU patients, 9% to 21% of the muscle fibers exhibited a complete loss of force‐generating capacity upon maximal calcium activation, despite displaying levels of atrophy comparable to those of force‐generating fibers at day 12 (Figure 1C), and similar protein coverage of myosin motor protein (Figure 2A). These muscle fibers from individuals exposed to 12 days of mechanical ventilation and immobilization were categorized into 12‐day force‐generating (“ICU_D12”) and 12‐day non‐force‐generating (“ICU_D12_NF”) muscle fibers.

### 3.2 Identification of Myosin PTMs Using LC–MS/MS‐Based Proteomics

To investigate the mechanisms underlying the partial and total decline in force generating capacity noted in fibers following 12 days of immobilization and mechanical ventilation, myosin protein PTMs were examined using LC–MS/MS‐based proteomics. The presence and absence of myosin PTMs were analyzed in TA muscle fibers from ICU_D1 and compared with the respective ICU_D12 force‐ and non‐force‐generating groups.

Twenty‐six muscle fibers across all experimental groups were included in this analysis. In humans, the TA muscle is predominantly composed of slow‐twitch fiber, with approximately 80% of the fibers expressing the βslow/type I myosin heavy chain (MyHC) isoform [17]. In line with this, all single muscle TA fibers included in this study predominantly expressed the type I MyHC isoform, although small amounts of fast MyHC isoforms (types IIa, IIx, and IIb) proteins were also detectable by mass spectrometry analysis (Figure 2A). Thus, the type I MyHC isoform dominated at the single muscle fiber level in the ICU_D1 and ICU_D12 groups, and there were no significant differences in MyHC isoform composition in the fibers analyzed in the three groups. Therefore, our PTM analyses were focused on the predominant type I MyHC isoform (Figure S1A). Overall, 35 PTMs of interest were identified, eight of which were common across conditions, while the other 27 PTMs were differentially regulated between the ICU_D12 and ICU_D1 groups (Table 1). These 27 modifications can be separated into four major classes: (1) Oxidation (“Oxi”, 68.6%); (2) Ubiquitination (“Ub”, 22.9%); (3) Acetylation (“Ac”, 5.7%); and (4) Methylation (“Methyl”, 2.8%) (Figure 2B,C). Ten PTMs (Lys86‐Oxi; Asp89‐Oxi; His97‐Oxid; Tyr162‐Oxi; Tyr164‐Oxi; His491‐Oxi; Phe494‐Oxi; Ans817‐Oxi; Ans923‐Oxi; and Asp1072‐Oxi) were added into ICU_D12 fibers (Figure S1B), with His97‐Oxid also found present in the ICU_D12_NF group, compared with the ICU_D1 control group. Furthermore, 17 PTMs were absent in the ICU_D12 groups compared with the ICU_D1, of which four PTMs (Lys598‐Ub; Lys1042‐Ub; Lys1073‐Ub; and Lys1870‐Ub) were absent in both ICU_D12, force‐ and non‐force‐generating fibers, while one modification (Asp1068‐Oxi) was found lacking only in the ICU_D12 group, compared with controls (Table 1). Strikingly, a total of 12 PTMs (Lys270‐Ub; Asn589‐Oxi; Asp752‐Oxi; His753‐Oxi; Lys757‐Methyl; Asn933‐Oxi; Ser1233‐Ac; Lys1316‐Ub; Ser1542‐Ac; Lys1569‐Ub; Asn1623‐Oxi; and Lys1895‐Ub) were exclusively absent in the ICU_D12_NF fibers group (Table 1).

**TABLE 1:** Identified post‐translational modifications in MyHC type I protein.

Position	Modification	Peptide sequence	Frequency (%)		
			ICU D1 control	ICU D12 force	ICU D12 non‐force
Present					
86	Oxidation	FDKIEDMAMLTFLHEPAVLYNLK	0	27^a^	0
89	Oxidation	FDKIEDMAMLTFLHEPAVLYNLK	0	36^a^	18
97	Oxidation	FDKIEDMAMLTFLHEPAVLYNLK	0	45^a^	27^a^
162	Oxidation	SEAPPHIFSISDNAYQYMLTDR	0	27^a^	0
164	Oxidation	SEAPPHIFSISDNAYQYMLTDR	0	36^a^	0
491	Oxidation	LQQFFNHHMFVLEQEEYKK	0	27^a^	0
494	Oxidation	LQQFFNHHMFVLEQEEYKK	0	36^a^	18
817	Oxidation	DSLLVIQWNIR	0	45^a^	9
923	Oxidation	VKEMNERLEDEEEMNAELTAK	0	27^a^	9
1072	Oxidation	LTQESIMDLENDKQQLDER	0	36^a^	0
Absent					
270	Ubiquitination	LASADIETYLLEKSR	50	27	0^b^
589	Oxidation	GKPEAHFSLIHYAGIVDYNIIGWLQK	25	45	9^b^
598	Ubiquitination	NKDPLNETVVGLYQK	50	0^b^	0^b^
752	Oxidation	LLSSLDIDHNQYK	25	36	0^b^
753	Oxidation	LLSSLDIDHNQYK	25	45	9^b^
757	Methylation	LLSSLDIDHNQYK	25	36	0^b^
933	Oxidation	VKEMNERLEDEEEMNAELTAK	25	45	9^b^
1042	Ubiquitination	LEQQVDDLEGSLEQEKK	50	9^b^	0^b^
1068	Oxidation	LTQESIMDLENDKQQLDER	25	9^b^	27
1073	Ubiquitination	LTQESIMDLENDKQQLDER	50	9^b^	0^b^
1233	Acetylation	LELDDVTSNMEQIIK	75	36	0^b^
1316	Ubiquitination	LTYTQQLEDLKR	50	27	0^b^
1542	Acetylation	MELQSALEEAEASLEHEEGK	25	54	18^b^
1569	Ubiquitination	AQLEFNQIKAEIER	25	36	0^b^
1623	Oxidation	MEGDLNEMEIQLSHANR	25	27	9^b^
1870	Ubiquitination	LQDLVDKLQLK	50	18^b^	0^b^
1895	Ubiquitination	QAEEAEEQANTNLSKFR	50	27	0^b^

^a^ Indicates post‐translational modifications with increased frequency compared with the control group.

^b^ Indicates post‐translational modifications with decreased frequency compared with control. The respective group's sample sizes are ICU_D1 control (*n* = 4), ICU_D12 force‐generating and non‐force‐generating (*n* = 11).^c^

^c^ The red colour letters highlighted in Table 1 indicate the modified peptide.

Noteworthy, among the 10 novel PTMs found in ICU_D12 fibers, seven were located within the myosin protein motor domain, one in the neck, and the other two in the rod domain. Moreover, among the five missing modifications, one was located in the motor domain, while the remaining four were missing in the rod domain. On the other hand, ICU_D12_NF fibers showed only one newly added modification located within the myosin motor domain compared with control ICU_D1 (Figure 2D).

### 3.3 Myosin Molecular Dynamics Simulations

**FIGURE 3:**
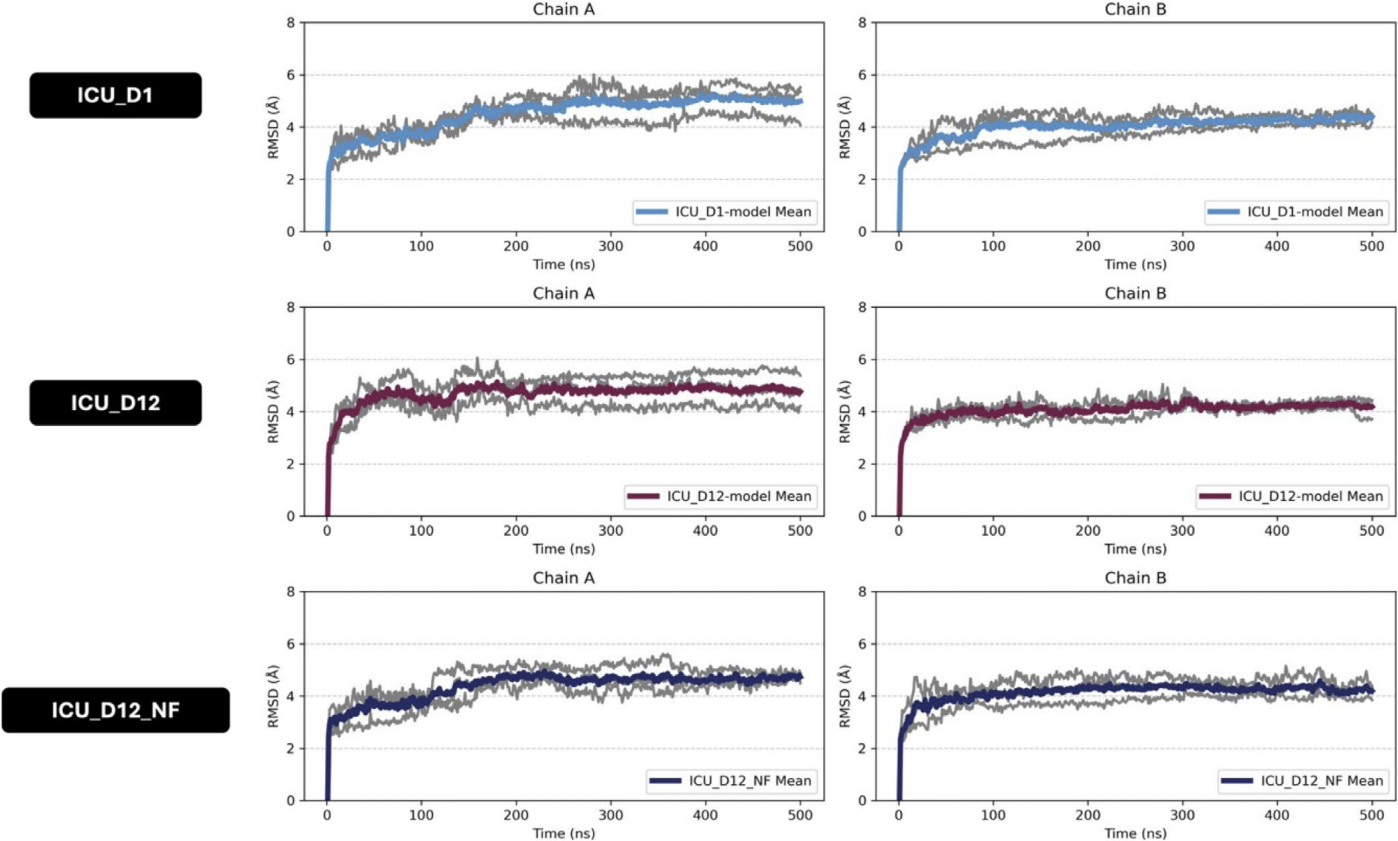
Backbone root‐mean‐square deviation (RMSD) profiles obtained from molecular dynamics (MD) simulations for systems ICU_D1, ICU_D12, and ICU_D12_NF. RMSD values (Å) were calculated relative to the initial structure to monitor structural stability and conformational changes over the 500 ns simulations. Gray lines represent individual simulation replicas, while colored lines indicate the mean RMSD across replicas. Results are shown separately for chains A and B.

**FIGURE 4:**
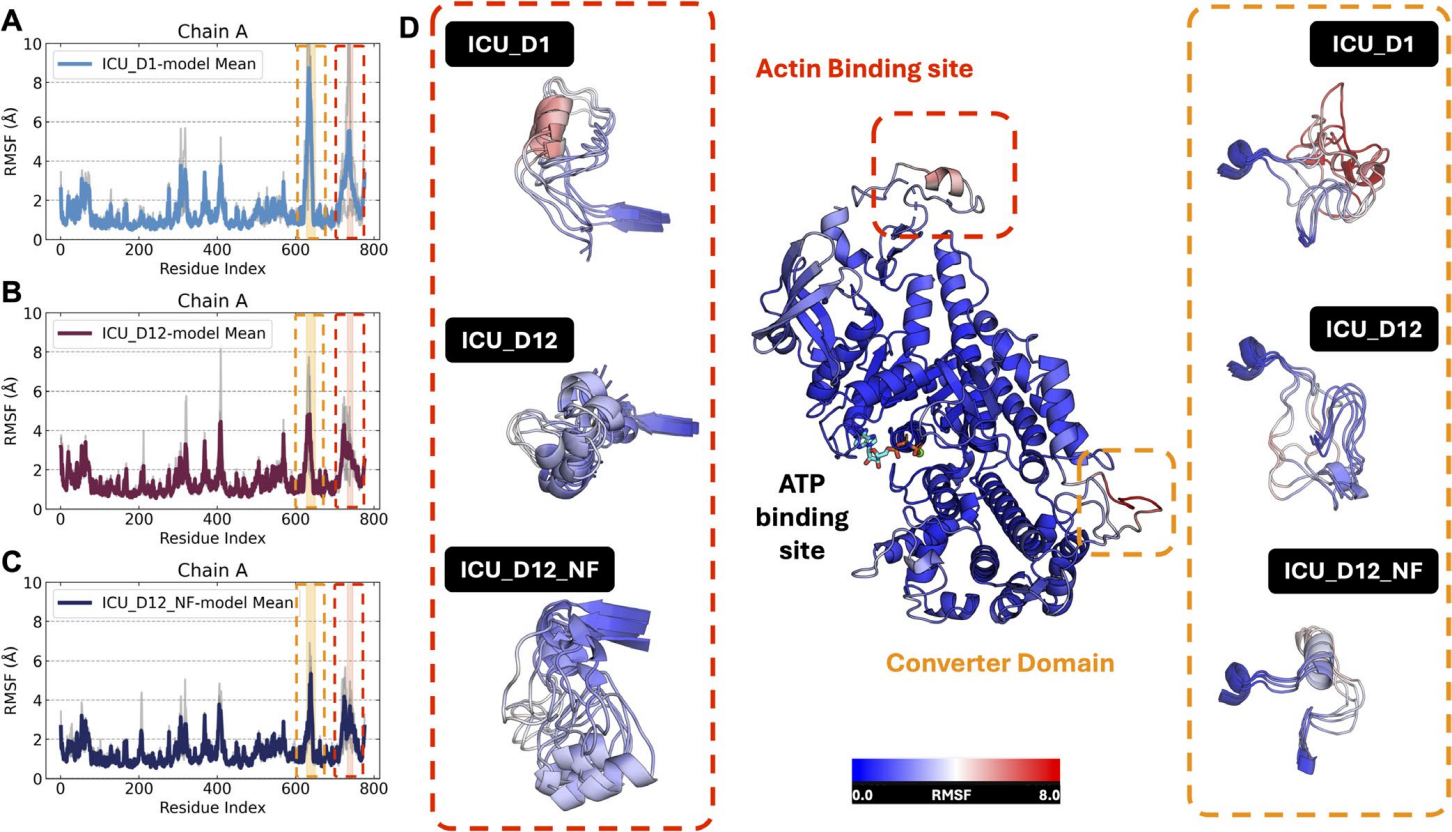
Prolonged mechanical ventilation and immobilization promote oxidative modifications and increased rigidity of myosin protein head. Residue RMSF values for each simulated replica (gray lines) and the corresponding mean RMSF (colored lines) for systems (A) ICU_D1, (B) ICU_D12, and (C) ICU_D12_NF, shown separately for chain A for clarity. (D) Molecular dynamics RMSF analysis confirms elevated flexibility of Actin‐binding and converter domains in the ICU_D1 control group, while after 12 days of ICU treatment (mechanical ventilation and immobilization) these critical domains required for myosin contractile function show decreased flexibility (i.e., rigidity).

Analysis of trajectories from the conventional MD simulations showed a clear difference in behavior between the three simulated systems (Figure 3). Specifically, ICU_D1, which in this research is used as a control, displayed notable flexibility in the regions defined as the converter domain (amino acid index positions 655–677) and the actin‐binding domain (amino acid index positions 757–771), with the latter being one of the most flexible regions (flexibility in these plots is characterized by the observation of larger amplitude fluctuations in given regions). Conversely, the main core of the myosin remained static (Figure S2). Notably, ICU_D12 and ICU_D12_NF exhibited reduced flexibility in both regions compared to ICU_D1 (Figure 4, again measured by the amplitude of the corresponding fluctuations in these regions), and this increased rigidity was particularly pronounced in chain A (Figures 3 and 4). Furthermore, visual inspection of these structures shows pronounced differences in loop conformations between these systems.

Both pathological systems, ICU_D12 and ICU_D12_NF, exhibited markedly reduced fluctuation amplitudes or RMSF values in the converter and actin‐binding domains relative to ICU_D1, indicating increased structural rigidity in these regions (Figure 4).

This reduction in flexibility was particularly pronounced for chain A (Figure 3). Importantly, all three systems were initiated from the same starting conformation. In addition, structural clustering and representative conformations extracted from the simulations (Figure 3) reveal substantial differences in loop conformations between ICU_D1 and the oxidized systems. These findings support the conclusion that oxidative modifications, rather than differences in initial geometry, reduce and alter local mobility in these functionally relevant regions.

**FIGURE 5:**
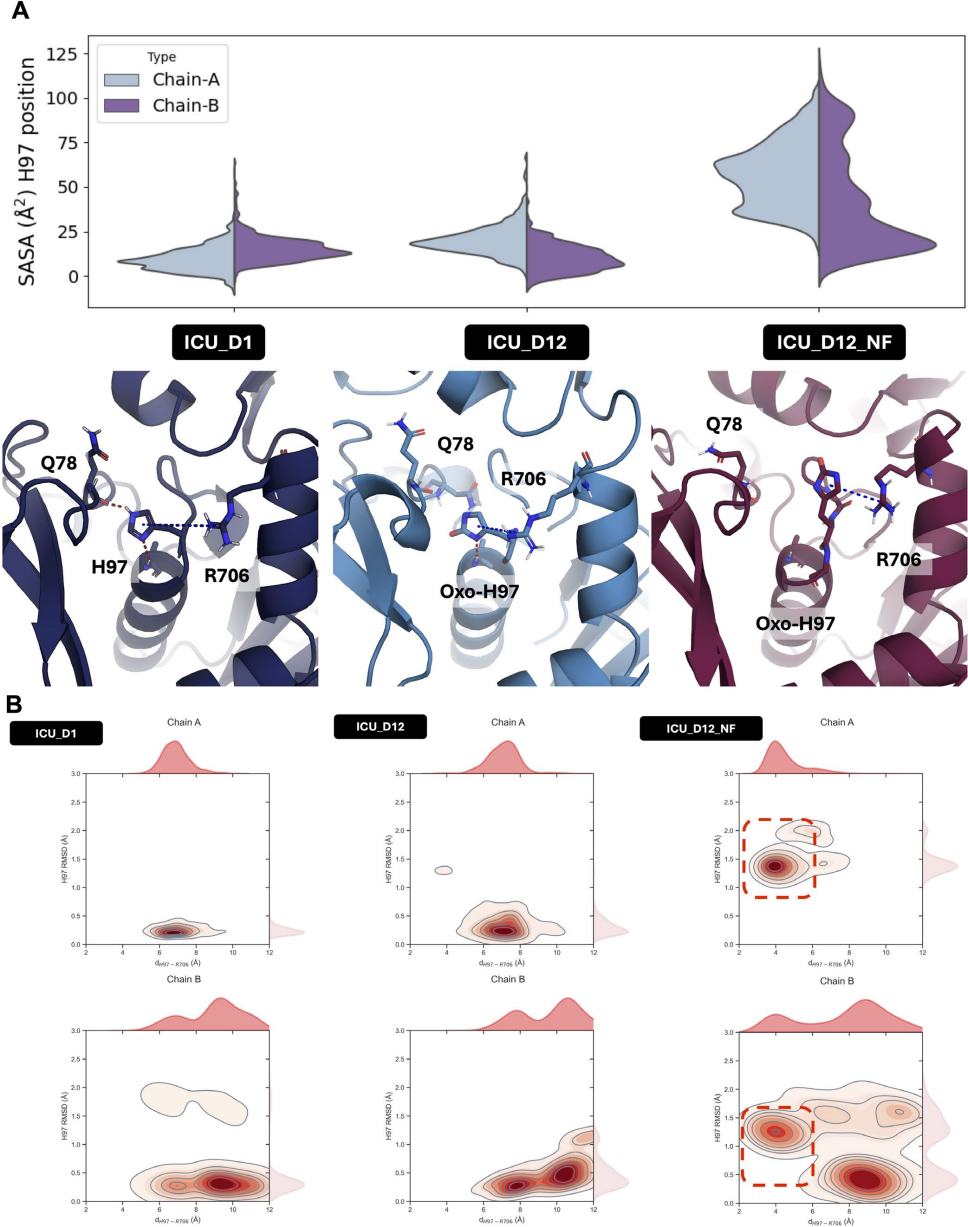
Structural instability and exposure of the myosin motor domain in non‐force‐generating fibers linked to H97 oxidation. (A) Solvent‐accessible surface area (SASA) distributions for residue H97 reveal that oxidized H97 (Oxo‐H97) in the ICU_D12_NF system is significantly more solvent‐exposed compared with ICU_D1 and ICU_D12, indicating reduced stability. Structural representations show that in ICU_D12_NF, H97 exhibits increased flexibility, frequently moving away from its native position and engaging in π–cation interactions with R706. This altered interaction may impair the conformational changes of the converter domain required for efficient powerstroke during contraction. (B) Kernel density estimates (KDE) correlating the RMSD of H97 with the distance between H97 and R706 across simulations of ICU_D1, ICU_D12, and ICU_D12_NF. ICU_D1 and ICU_D12 exhibit compact distributions consistent with stable positioning of H97, whereas ICU_D12_NF displays broader, multimodal distributions. The red dashed regions highlight a stable π–π interaction unique to the ICU_D12_NF system, further supporting the distinct structural signature of non‐force‐generating fibers.

To further explore this issue, we performed detailed structural analysis of position His97, which is located at the N‐terminal region of helix‐6 (Figure 5). This analysis showed different behavior in the three groups. In ICU_D1, the non‐oxidized histidine remained stable by keeping the two hydrogen bonds within the backbone of Ala100 and Gln78. In ICU_D12, His97 is oxidized to oxo‐histidine, and despite the loss of the hydrogen bond with Gln78, the modified amino acids remained in the same position. However, in ICU_D12_NF fibers, this oxo‐histidine was not stable and tended to be exposed to the solvent (Figure S4). Accordingly, Solvent Accessible Surface Area (SASA) analysis allowed us to monitor this exposure, and the ICU_D12_NF group exhibited a more solvent‐exposed His97 (Figure 5A). In this system, the oxo‐histidine was particularly more flexible and tended to interact with the side chain of Arg706 (Figure 5B, Figure S4).

While the role of histidine in forming π‐π, cation‐π, and CH‐π interactions in proteins is well understood [18], the nature of the corresponding interactions involving an oxidized histidine (2‐oxo‐hisitine or oxo‐his) remains an under‐explored area in chemical biology [19]. Specifically, while the aromatic imidazole ring of histidine can readily participate in π‐π and cation‐π interactions, oxidation at the C2‐position alters the electronic distribution within the ring, perturbing the canonical π‐cloud. As a result, the ring may no longer sustain a typical cation‐π interaction, although electrons may still be delocalized (Figure S8). This partial delocalization allows for possible dipolar and electrostatic interactions involving this histidine. Furthermore, the introduction of an electron‐withdrawing carbonyl at position C2 increases the local electronegativity of the ring system, potentially enhancing electrostatic interactions with nearby positively charged residues, such as in our case, the Arg706.

Consistent with the above observations, our MD simulations showed persistent proximity between oxo‐His97 and Arg706 in the non‐force‐generating fiber (ICU_D12_NF), suggesting the presence of an altered yet potentially stabilizing electrostatic or dipole‐cation contact between these side chains. We note, however, that due to the limited experimental and theoretical literature available on the physicochemical behavior of oxo‐His in‐protein environments, these observations should be considered hypothesis‐generating and encourage future work into the potential interaction of this modified residue [20, 21].

Finally, to better understand the local behavior of the Arg706 side chain across different oxidation states, we analyzed the time‐evolution of the RMSD of this residue over the course of the simulations of different systems (Figure 3). Our results show that this side chain remains relatively stable in the ICU_D12 system, which carries multiple oxidations, including 2‐oxo‐His97 (H97ox). In contrast, side chain fluctuations are more pronounced in the ICU_D1 system, which lacks these additional oxidative modifications. Further, although H97 is oxidized in both ICU_D12 and ICU_D12_NF systems, a persistent interaction between oxo‐His97 and Arg706 side chains is observed only in ICU_D12. In the ICU_D12_NF system, this interaction is transient and more flexible, as confirmed by the increased RMSD of Arg706, as well as by visual inspection (Figure S4). This suggests that the redox state of nearby residues in ICU_D12 contributes to a more rigid conformation, effectively stabilizing R706 and allowing for a consistent interaction with the negatively polarized oxo‐His97.

Taken together, these results indicate consistency across replicas (Figures S6 and S7) highlighting how multisite oxidation may restrict local flexibility, reinforcing the notion of redox‐mediated allosteric regulation. While it is true that oxidation alters the classical aromatic and electronic properties of histidine, it does not necessarily abolish its ability to engage in specific non‐covalent or even covalent interactions. In fact, literature evidence supports that 2‐oxo histidine can exhibit electrophilic character and form covalent adducts with nucleophiles, especially thiols and amines in biological contexts [22]. In the specific case of ICU_D12_NF, the oxo‐His residue is more exposed to the solvent, which may facilitate covalent attachment to other adducts or potentially promote intramolecular cross‐linking within the same myosin molecule.

## 4 Discussion

Critically ill patients undergoing prolonged mechanical ventilation and immobilization rapidly develop skeletal muscle wasting and weakness [23, 24, 25]. Muscle biopsies from ICU patients reveal significant ultrastructural changes, including impaired thick filament organization and preferential myosin loss [2, 26, 27, 28, 29]. However, the molecular mechanisms underlying myosin contractile dysfunction and loss remain unknown. Herein, we examined the effects of prolonged ICU treatment (i.e., mechanical ventilation and immobilization) on myosin post‐translational modifications and their role in muscle weakness. Limb muscle biopsies from a previous study [2] collected from neuro‐ICU patients were reanalyzed with proteomic and molecular dynamics simulation analyses to assess myosin post‐translational modifications and their impact on protein structure and function in relation to muscle fiber size and specific force on the 12th compared with the 1st day of immobilization and mechanical ventilation. Twelve days of mechanical ventilation and immobilization had a strong negative effect on muscle fiber size and specific force [2] coupled to post‐translational myosin modifications. Abnormal post‐translational modifications (e.g., oxidation, ubiquitination, acetylation, and methylation) were predicted by MD simulations to increase myosin protein rigidity and contractile dysfunction. Additionally, a subset of fibers exhibited complete loss of contractility, showing a unique proteomic signature characterized by much fewer post‐translational modifications, predicted to destabilize myosin motor domain structure and function.

Elevated oxidative stress is known to impair skeletal muscle contractile function [30]. In addition, in vitro and in vivo studies show that oxidative modifications induce sarcomere protein damage due to altered protein ultrastructure, protein–protein binding interaction, and calcium sensitivity [31]. In parallel with the decline in specific force after 12 days of immobilization and mechanical ventilation, multiple myosin post‐translational modifications were observed, with oxidation being the most prevalent one, accounting for over 60% of all identified modifications. Prolonged mechanical ventilation and immobilization are associated with inflammation and mitochondrial dysfunction, both known to enhance reactive oxygen species (ROS) production in critically ill patients [32]. Our previous experimental studies confirm that long‐term mechanical ventilation and immobilization ROS‐induced modifications (e.g., oxidation and carbonylation) impair efficiency, motility speed, and force‐generation capacity of myosin at the motor protein level [4, 5, 33]. Thus, mechanisms underlying muscle dysfunction in ICU patients with CIM are multifactorial and not only related to muscle fiber atrophy, altered membrane excitability and excitation‐contraction coupling, and preferential myosin loss. Herein, we additionally suggest that myosin post‐translational modifications are likely linked to compromised muscle function and also in the development of non‐force‐generating fibers.

ICU patients' myofibers exhibit increased oxidative stress‐induced myosin post‐translational modifications [6, 27], but how these modifications disturb myosin structure and contractile function remains unclear. Observations from the current study suggest increased rigidity of the myosin motor domain in response to oxidative modifications after 12 days of ICU treatment, affecting myosin actin‐binding and converter domains. Structural analysis indicates proximity between oxidized residues and methionine residues (Figure S5), suggesting that methionine oxidation may trigger the oxidative process, as previously described [34, 35]. Recent data suggest an increased fraction of myosin protein trapped in the super‐relaxed state (i.e., blocked myosin‐actin binding interaction) [36]. However, this phenomenon was uniquely observed in the diaphragm and thus unlikely to explain the limb muscle weakness observed herein. Conversely, myosin oxidative modifications have been noticed in both respiratory and limb muscles following prolonged mechanical ventilation [4, 6, 27]. Thus, oxidized sites located close to each other in the converter domain are suggested to affect the myosin lever arm movement and decrease myosin head flexibility, ultimately impairing myosin function in ICU patients.

In this study, we focused on myosin post‐translational modifications since the preferential myosin loss is considered a hallmark of CIM [1]. However, parallel changes in post‐translational modifications of other contractile proteins contributing to the loss in muscle function cannot be completely ruled out. Our previous analyses of the regulation of muscle contraction at both the cellular and motor protein levels (by a single muscle fiber in vitro motility assay where myosin is extracted from single muscle fiber segments) have shown similar changes in force‐generating capacity at the cell and motor protein levels. Analyses at the motor protein level were conducted using the same actin purified from rabbit skeletal muscle and rhodamine‐phalloidin labelling, following myosin extraction from both control and long‐term mechanically ventilated immobilized muscle. In addition, the restorative effects of the chaperone co‐inducer BGP‐15 on force‐generating capacity were consistent at both the cellular and motor protein levels, adding further support of the dominant role of myosin post‐translational modifications in regulating muscle function [5, 6].

Prolonged mechanical ventilation leads to the emergence of non‐force‐generating fibers, which might contribute to muscle weakness severity in critically ill patients. We previously reported in a time‐course experimental study a progressive increase of this subset of fibers, from 7% to 37%, in the diaphragm after 6 h to 14 days of mechanical ventilation and immobilization [4] and confirmed in a prospective clinical study showing 9% to 21% non‐force‐generating limb muscle fibers in 5 of 10 neuro‐ICU patients exposed to 10–12 days mechanical ventilation and immobilization [2]. In this study, non‐force‐generating myosin expressing fibers had similar fiber CSA as in force‐generating ICU D12 fibers. However, these dysfunctional fibers uniquely exhibited increased solvent‐accessible surface area at oxidized histidine 97, suggesting higher solvent exposure, pointing to a structural rearrangement upon oxidation. While increased solvent exposure does not inherently imply reduced structural stability of the myosin head, detailed analysis reveals a newly reinforced π–cation interaction between His97 and Arg706, observed exclusively in the non‐force‐generating fiber.

Interestingly, this particular histidine oxidation was also detected in fast‐twitch fibers (type IIx) in the diaphragm of rats exposed to 5 days of mechanical ventilation [37]. This particular histidine (i.e., H98) was also found oxidized in the detectable fraction of myosin type IIx protein from non‐force‐generating fibers (Table S1, Figure S9). One may envision that oxidized histidine residues may serve as electrophiles and readily react with nearby nucleophiles to form covalent adducts, leading to the complete loss of the lever arm movement and explaining the complete loss of myosin contractile function observed in ICU_D12_NF fibers. This aligns with previous evidence demonstrating that exacerbated and cumulative oxidative stress may trigger the transition of cross‐bridges from the force‐generating to non‐force‐generating state [38]. Hence, we propose that non‐force‐generating fibers represent a subpopulation of fibers likely in an advanced degenerative state resulting from the cumulative oxidative damage induced by prolonged mechanical ventilation and immobilization.

Although oxidation was predominant, other identified post‐translational modifications may also affect myosin in ICU patients. In this context, protein ubiquitination plays a crucial role in several biological processes (e.g., muscle proteostasis, mitophagy, and regeneration) [39, 40]. We found eight ubiquitinated sites in the myosin molecule of ICU_D1 controls, while after 12 days, four ubiquitinated sites were lacking in ICU_D12 fibers, mostly in the rod domain. Remarkably, all identified ubiquitinated sites were found missing in non‐force‐generating fibers. To the best of our knowledge, this is the first report of altered ubiquitylation of human myosin muscle protein following long‐term ICU treatment. Previous studies have shown increased activation of some ubiquitin‐proteasome pathway markers in critically ill patients undergoing thoracic surgery, thought to contribute to muscle atrophy in mechanically ventilated patients [41]. Concerning this, however, our proteomic supports that these contractile dysfunctional fibers expressed the full‐length myosin protein (i.e., 223 kDa), suggesting the protein peptide sequence integrity in these biopsies. In our view, these altered ubiquitinated sites are most likely mono‐ubiquitination, which are highly dynamic and commonly involved with the regulation of enzymatic activity rather than protein degradation [42, 43]. Thus, we envision that these lost ubiquitinations could negatively impact myosin structure and function in long‐term mechanically ventilated and immobilized ICU patients.

Acetylation represents another class of post‐translational modifications that regulate muscle contraction in health and disease [44]. We identified two lacking acetylation (Ser1233‐Ac and Ser1542‐Ac) in the myosin rod domain of myosin in non‐force‐generating fibers. Similar acetylation modifications in the myosin rod domain have been reported in previous experimental and clinical ICU studies [27, 37]. Decreased acetylation of contractile proteins has also been reported as a hallmark in disuse‐induced muscle wasting and weakness [45]. In line with this, histone deacetylases (HDACs), particularly HDAC4, have been demonstrated to target the myosin‐heavy chain and promote muscle dysfunction [46]. Missing acetylation sites in the myosin rod domain can disrupt thick filament stability, myosin head positioning, and the ATPase turnover, contributing to muscle dysfunction in diseases such as congenital myopathies and heart failure [10, 47]. Therefore, the loss of acetylation in the myosin rod domain may contribute to the impaired myofibers' force generation capacity in critically ill patients.

Protein methylation also controls important biological processes [48]. Here, we identified a missing methylation (C‐term K757) from the myosin motor domain of non‐force‐generating fibers. Previously, our group reported myosin methylation changes in both experimental and clinical ICU studies [6, 27]. These modifications have been suggested to be associated with impaired myosin filament stability, assembly, and motility, similar to aging‐related methylation myosin modifications in humans [49]. Furthermore, we have previously shown that BGP‐15 treatment protects the soleus muscle strength in parallel with mitigating the methylation of the myosin rod domain in rats subjected to prolonged mechanical ventilation and immobilization [6]. However, the understanding of the impact of methylation on myosin structure and function remains incomplete. Overall, the altered methylation in the myosin head (e.g., K757‐methylation) may contribute to the loss of myosin function in non‐force‐generating fibers.

Study limitations: (1) Our patient sample size (*n* = 6) and the number of muscle fibers (*n* = 26) analyzed are relatively small due to the challenge of obtaining intra‐subject matched muscle biopsies in long‐term ICU patients. However, to our knowledge, this is the first clinical study that provides mechanistic insights into how myosin post‐translational modifications might negatively affect skeletal muscle contractile function in long‐term (12 days) mechanically ventilated ICU patients. Furthermore, all muscle samples were collected from neuro‐ICU patients with relatively similar clinical conditions (i.e., central nervous system injury) without underlying chronic disease and intrasubject time‐course comparisons (ICU day 12 vs. day 1), minimizing the inherent variability between subjects' responses to long‐term ICU treatment. (2) Other myofibrillar proteins besides the molecular motor protein myosin also affected by post‐translational modifications may also influence force‐generation capacity. However, myosin‐actin interactions represent the final functional contractile unit and represent a key factor in the motor system. (3) Our molecular dynamics simulations are based on the myosin crystal structure PDB ID: 4DB1, NIH 3D. (2025). *Cardiac human myosin S1dC, beta isoform complexed with Mn‐AMPPNP* (Version 1.x). NIH 3D (https://doi.org/10.60705/3DPX/22248.1) that contains some unresolved structural loops, and computational reconstruction of these missing loops remains uncertain, relying on predicted conformations rather than structural data. However, we note that despite our homology modeling process with Modeler generating multiple loop conformations, all three systems (ICU_D1, ICU_D12 and ICU_D12_NF) share the same underlying loop structural scaffold in our starting simulations, and thus any observed differences in RMSD and RMSF cannot stem from variations in starting loop conformations [50]. Our MD simulations show that both oxidized systems (ICU_D12 and ICU_D12_NF) exhibit reduced loop mobility and flexibility compared with ICU_D1. These findings are consistent across independent simulation replicates (Figures 3 and 4, Figures S3–S7), further supporting the reproducibility of the observed effect. In summary: (1) All three models began with equivalent loop conformations; (2) Only oxidized systems exhibit dampened loop motions; (3) This strongly suggests that the oxidation pattern, not loop bias, underlies the reduced dynamics. These insights reinforce our main conclusion: that site‐specific redox modifications, particularly in residues near the loop region, impact the mechanical properties of myosin. (4) The crystal structure used for the myosin simulations is of the dimeric form of myosin, which may not fully represent the physiological organization of myosin heads in the thick filament or the interacting‐head motif (IHM). However, the use of dimeric structure allows us to capture potential inter‐chain and interface‐mediated effects, which are particularly relevant in the context of oxidative modifications (see e.g., refs. [51, 52, 53, 54], among others). Several of the oxidized residues analyzed in this work are positioned near regions that may be influenced by inter‐chain proximity, and the dimeric model allows us to explore how redox perturbations propagate through local interaction networks and affect allosteric communication within the motor domain. The observed changes in flexibility, solvent exposure, and residue‐residue interactions are consistent with mechanisms previously proposed using the same structural framework, lending further support to the validity of our conclusions [10, 55, 56, 57].

## 5 Conclusions

Abnormal post‐translational myosin modifications (e.g., oxidation, ubiquitination, acetylation, and methylation) are associated with muscle weakness in neuro‐ICU patients. Our findings suggest that oxidative modifications are predicted to increase myosin head rigidity, affecting the actin‐binding and converter domains, impairing myosin function. Moreover, a subset of non‐force‐generating fibers exhibited a unique proteomic signature predicted to enhance instability and rigidity of the myosin protein motor domain. This development of non‐force‐generating fibers likely exacerbates muscle weakness in critically ill patients. These novel findings may provide a rationale for the design of novel therapies targeting myosin structure and contractile function preservation in long‐term mechanically ventilated ICU patients.

## Author Contributions

**Anna Widgren:** formal analysis. **Fernando Ribeiro:** writing – original draft, formal analysis, writing – review and editing. **Jonas Bergquist:** writing – original draft, formal analysis, methodology, resources. **Yvette Hedström:** methodology, formal analysis, investigation. **Nicola Cacciani:** formal analysis, investigation. **Lars Larsson:** conceptualization, investigation, writing – original draft, writing – review and editing, methodology, formal analysis, supervision, project administration, resources. **Peter M. Kasson:** conceptualization, formal analysis, writing – original draft, writing – review and editing, methodology, resources. **Anselmo S. Moriscot:** supervision, writing – review and editing. **Shina C. L. Kamerlin:** conceptualization, writing – original draft, writing – review and editing, formal analysis, methodology, resources. **Bruno Di Geronimo:** writing – original draft, formal analysis, methodology, writing – review and editing.

## Funding

FR was supported by São Paulo Research Foundation (FAPESP grant 2022/14495‐0). ASM was supported by FAPESP grant 2025/00791‐5 and the National Council for Scientific and Technological Development (CNPq 305494/2022‐8). PMK was supported by WAF 2020.0209 from the Knut and Alice Wallenberg Foundation. SCLK and BDG were supported by start‐up funds from the Georgia Institute of Technology. SCLK is the Georgia Research Alliance—Vasser Woolley Chair of Molecular Design at the Georgia Institute of Technology. The computation simulations were enabled by resources provided by the National Academic Infrastructure for Supercomputing in Sweden (NAISS), partially funded by the Swedish Research Council through grant agreement no. 2024‐0816 and 2022‐06725. We also acknowledge NAISS for awarding this project access to the LUMI supercomputer, owned by the EuroHPC Joint Undertaking, hosted by CSC (Finland) and the LUMI consortium. This work also used the HIVE supercomputer cluster, which is supported by the National Science Foundation under grant number 1828187. This research was supported in part through research cyberinfrastructure resources and services provided by the Partnership for an Advanced Computing Environment (PACE) at the Georgia Institute of Technology, Atlanta, Georgia, USA. LL received financial support from the Swedish Medical Research Council (8651), Stockholm City Council (Alf 20150423, 20170133), ESICM, and Viron MMI.

## Ethics Statement

The study was approved by the ethics committee at the Karolinska Hospital (Dnr 2016/242‐31/2).

## Consent

Written consent was obtained from the patients' close relatives.

## Conflicts of Interest

The authors declare no conflicts of interest.

## Supporting information


**Figure S1:** Structural representation of human β‐cardiac myosin heavy chain (MYH7). (A) Overall MYH7 structure (PDB ID: 4DB1), showing Chain A in cartoon representation (purple) and Chain B as a surface (tan). The ATP molecule (green sticks) and coordinated water molecules are highlighted in the binding pocket. (B) Detailed view of MYH7 Chain A in cartoon representation, with amino acid residues analyzed for oxidative post‐translational modifications (red sticks). Key modified positions are labeled, including catalytic and structural residues (e.g., H97, H491, D89, Y162/Y164, N589, D752/H753, and F494). The ATP molecules are shown in green sticks.
**Figure S2:** Clustering representation of the ICU_D1, ICU_D12, and ICU_D12_NF models over the last 400 ns of the simulations following 100 ns of equilibration. Representative cluster structures are colored according to their RMSF values, with deep blue indicating more rigid regions and red denoting higher flexibility.
**Figure S3:** Differential root mean square fluctuation (ΔRMSF) profiles of MYH7 models under distinct ICU conditions. Residue‐wise ΔRMSF values (in Å) are shown for comparisons between the ICU_D1 model and either ICU_D12 (top panels) or ICU_D12_NF (bottom panels), displayed separately for Chain A (left) and Chain B (right). Positive values indicate residues with increased flexibility in ICU_D1 relative to the comparison model, while negative values indicate decreased flexibility. The largest fluctuations are observed within the C‐terminal tail and converter regions (residues ~650–780), with additional smaller peaks in the motor domain (residues ~1–400). These results suggest that structural dynamics are differentially affected across functional domains, particularly in regions critical for force transmission and conformational transitions.
**Figure S4:** Representative conformations of MYH7 under different ICU models, highlighting structural changes around Histidine 97. Representations from molecular dynamics (MD) simulations are shown for ICU_D1 (left), ICU_D12 (middle), and ICU_D12_NF (right). Key residues in the interaction network: Q78, H97 (or its oxidized form, oxo‐H97), and R706 are displayed in stick representation. In the ICU_D1 model, H97 maintains native contacts with neighboring residues, while in ICU_D12 and ICU_D12_NF models, oxidation of H97 alters its orientation and interactions, particularly with R706. These changes suggest that oxidative modification at H97 may destabilize local residue contacts and promote distinct conformational states that might contribute to impaired myosin motor function.
**Figure S5:** Structural context of oxidizable residues in Myosin MYH7 (Chain A, PDB ID: 4DB1). The overall protein structure is shown in cartoon representation, with selected amino acids displayed in stick format to emphasize their spatial relationships. Panels highlight the proximity of potentially oxidizable residues to nearby methionines or aromatic residues: (A) Lysine 86 near Methionine 113; (B) Aspartic acid 89 adjacent to Methionine 92; (C) Histidine 97 close to Methionine 77; (D) Tyrosines 162 and 164 in proximity to Methionine 165; (E) Histidine 491 and Phenylalanine 494 near Methionine 515; (F) Aspartic acid 752 and Histidine 753 adjacent to Methionine 776. These panels provide a structural basis for evaluating potential oxidative interactions within the myosin motor domain.
**Figure S6:** Time evolution of the distance between Histidine 97 and Arginine 706 over the last 400 ns of the simulations for systems ICU_D1, ICU_D12, and ICU_D12_NF. Histidine 97 is oxidized in the ICU_D12 and ICU_D12_NF systems.
**Figure S7:** Root‐mean‐square deviation (RMSD) analysis of Arginine 706 in ICU_D1 and ICU_D12 calculated using all heavy atoms (non‐hydrogen atoms) relative to the minimized starting structure immediately prior to the production MD phase (0 ns). Three independent replicas are shown in gray, with the corresponding mean values shown in color.
**Figure S8:** Schematic 2D representations of amino acid side chains and their proposed oxidative modifications. Shown are representative amino acids susceptible to oxidative post‐translational modifications, including histidine, asparagine, tyrosine, phenylalanine, aspartic acid, and lysine. For each residue, the unmodified side chain (left) and potential oxidized states (right) are depicted, highlighting the chemical changes (e.g., hydroxylation, carbonylation, or other oxygen‐containing adducts). Atoms are color‐coded (black for carbon, blue for nitrogen, red for oxygen, and yellow for sulfur) to emphasize structural alterations upon oxidation.
**Figure S9:** Schematic representation of post‐translational modifications in type IIx myosin. A 2D schematic illustrates the identified post‐translational modifications and their positions within the myosin structure for the ICU_D1, ICU_D12, and ICU_D12_NF groups. The diagram highlights group‐specific modification patterns, providing a comparative view of structural changes associated with different ICU conditions.
**Table S1:** Identified post‐translational modifications in MyHC type IIx protein.

## Data Availability

The data that support the findings of this study are available on request from the corresponding author. The data are not publicly available due to privacy or ethical restrictions.
