# Supplementary material for: Myosin Post‐Translational Modifications Associated With Critical Illness Myopathy
Source: Acta Physiol (Oxf). 2026 Jun 18;242(7):e70240. doi: 10.1111/apha.70240 (PMC13279842; doi:10.1111/apha.70240)
Supplement: Supplementary file 1 — Figure S1: Structural representation of human β‐cardiac myosin heavy chain (MYH7). (A) Overall MYH7 structure (PDB ID: 4DB1), showing Chain A in cartoon representation (purple) and Chain B as a surface (tan). The ATP molecule (green sticks) and coordinated water molecules are highlighted in the binding pocket. (B) Detailed view of MYH7 Chain A in cartoon representation, with amino acid residues analyzed for oxidative post‐translational modifications (red sticks). Key modified positions are labeled, including catalytic and structural residues (e.g., H97, H491, D89, Y162/Y164, N589, D752/H753, and F494). The ATP molecules are shown in green sticks. Figure S2: Clustering representation of the ICU_D1, ICU_D12, and ICU_D12_NF models over the last 400 ns of the simulations following 100 ns of equilibration. Representative cluster structures are colored according to their RMSF values, with deep blue indicating more rigid regions and red denoting higher flexibility. Figure S3: Differential root mean square fluctuation (ΔRMSF) profiles of MYH7 models under distinct ICU conditions. Residue‐wise ΔRMSF values (in Å) are shown for comparisons between the ICU_D1 model and either ICU_D12 (top panels) or ICU_D12_NF (bottom panels), displayed separately for Chain A (left) and Chain B (right). Positive values indicate residues with increased flexibility in ICU_D1 relative to the comparison model, while negative values indicate decreased flexibility. The largest fluctuations are observed within the C‐terminal tail and converter regions (residues ~650–780), with additional smaller peaks in the motor domain (residues ~1–400). These results suggest that structural dynamics are differentially affected across functional domains, particularly in regions critical for force transmission and conformational transitions. Figure S4: Representative conformations of MYH7 under different ICU models, highlighting structural changes around Histidine 97. Representations from molecular dynamics (MD) sim [file APHA-242-e70240-s001.zip › Supplementary Table E1_separate-file_fig+legend_20260424_FR.docx]

**Table E1**. Identified post-translational modifications in MyHC type IIx protein.

|  |  |  | **Frequency (%)** | | | |
| --- | --- | --- | --- | --- | --- | --- |
| **Position** | **Modification** | **Peptide Sequence** | **ICU D1**  **Control** | **ICU D12**  **Force** | | **ICU D12**  **Non-force** |
| **Present** |  |  |  |  |  | |
| 98 | Oxidation | YDKIEDMAMMTHL**H**EPAVLYNLK | 0 | 0 | 28^a^ | |
| 494 | Oxidation | LQQFFN**H**HMFVLEQEEYKK | 0 | 33^a^ | 0 | |
| 497 | Oxidation | LQQFFNHHM**F**VLEQEEYKK | 0 | 44^a^ | 28^a^ | |
| **Absent** |  |  |  |  |  | |
| 154 | Oxidation | RQEAPP**H**IFSISDNAYQFMLTDR | 33 | 22^b^ | 0^b^ | |
| 165 | Oxidation | RQEAPPHIFSISDNAYQ**F**MLTDR | 33 | 22^b^ | 14^b^ | |
| 273 | Ubiquitination | LASADIETYLLE**K**SR | 66 | 22^b^ | 0^b^ | |
| 601 | Ubiquitination | N**K**DPLNETVVGLYQK | 66 | 0^b^ | 0^b^ | |
| 1046 | Ubiquitination | LEQQVDDLEGSLEQE**K**K | 66 | 11^b^ | 0^b^ | |
| 1200 | Oxidation | **H**ADSVAELGEQIDNLQR | 33 | 0^b^ | 0^b^ | |
| 1541 | Oxidation | QVEQE**K**SELQAALEEAEASLEHEEGK | 66 | 22^b^ | 42 | |
| 1635 | Oxidation | MEGDLNEMEIQLN**H**ANR | 33 | 22^b^ | 0^b^ | |

Supplementary Table E1. Identified post-translational modifications in MyHC type IIx protein. aIndicates post-translational modifications with increased frequency compared with the control group; bIndicates post-translational modifications with decreased frequency compared with control. The respective group’s sample sizes are ICU_D1 control (n = 4), ICU_D12 force-generating (n = 11) and ICU_D12 no force-generating fibers (n = 11).
